# Primary Sclerosing Cholangitis: Palliative Surgery or Transplantation?

**DOI:** 10.1155/1992/57190

**Published:** 1992

**Authors:** John L. Cameron

**Affiliations:** The Johns Hopkins University School of Medicine Richard Starr Ross Research Building, 720 Rutland Avenue, Room 759 Baltimore Maryland 21205-2196 USA


*HPB Surgery*, 1992, Vol. 5, pp. 275–290
Reprints available directly from the publisher
Photocopying permitted by license only

© 1992 Harwood Academic Publishers GmbH
Printed in the United Kingdom

### HPB INTERNATIONAL

EDITORIAL & ABSTRACTING SERVICE

JOHN TERBLANCHE, EDITOR
Department of Surgery,
Medical School · Observatory 7925
Cape Town · South Africa
Telephone: (021) 47-1250 Ext. 2
Telefax (021) 448-6461

### PRIMARY SCLEROSING CHOLANGITIS: PALLIATIVE SURGERY OR TRANSPLANTATION?

### ABSTRACT

*Ismail, T., Angrisani, L., Powell, J.E., Hubscher, S., Buckels, J., Neuberger, J., Elias, E., McMaster, P. (1991) Primary sclerosing cholangitis: surgical options, prognostic variables and outcome. British Journal of Surgery; 78 564–567*

**The natural history of primary sclerosing cholangitis (PSC) is poorly defined and its management remains controversial. Forty-eight symptomatic patients (median age 39 years, range 8-67 years; 30 male) with PSC were reviewed retrospectively. Thirty patients had infllammatory bowel disease. Four patients (8 per cent) developed or had an associated malignancy. Twenty-one (44 per cent) died; overall 5 year actuarial survival was 30 per cent. Twenty-three patients had 27 non-transplant related biliary operations (16 patients specifically for PSC of whom 12 died. Serum bilirubin was the only parameter to improve after biliary surgery. Seventeen patients (35 per cent) underwent orthotopic liver transplantation (OLT) of whom nine are currently alive (1 year projected survival of 55 per cent). Previous biliary surgery correlated with a poor outcome ($P<0.0001$) after OLT. Being male, presence of cirrhosis, duration of symptomatic disease (> 3 years) and a serum bilirubin level > 100 μmol/1 at presentation, were independently associated with a poor outcome ($P<0.05$). These data provide evidence that PSC is a progressive disease and conventional surgical options have little influence on the outcome. Previous biliary surgery adversely affects outcome following OLT. For progressive liver disease, liver transplantation should be considered the treatment of choice.**

### PAPER DISCUSSION

KEY WORDS: Sclerosing cholangitis, liver transplant, hepaticojejunostomy

Primary sclerosing cholangitis is an idiopathic disease, with an extremely broad clinical spectrum. Because of this broad spectrum, most published series, which contain only a modest number of patients because of the rarity of the disorder, give a somewhat skewed outlook of the natural history. For instance, there are some series that suggest the disease runs an exceedingly benign course,[1] with the vast majority of patients surviving five years without therapy. Other series, including the group of 48 patients presented by Ismail *et al.*, demonstrate a five year survival of only 30%. The reason for these disparities is that each series consists of selected patients. The means of selection determine the subsequent clinical course. Only when one institution accumulates a large enough clinical experience, or many smaller series from a variety of institutions are combined, will we accurately understand the natural history of this disorder.

The current series consists of 48 symptomatic patients, a substantial number of whom had far advanced liver disease. Presenting features included variceal hemorrhage in 21%, and encephalopathy in 25%. Histology was available from 45 of their patients, and in 23, or 51%, established cirrhosis was present. Thus this series consists of patients with well advanced sclerosing cholangitis, many of whom would not be candidates for any other therapeutic intervention except liver transplantation.

Therefore, their conclusion that conventional surgical options have little influence on the outcome is inevitable, since to benefit from conventional surgical therapy only mild to moderate liver disease can be present. From our institution,[2] as well as from others,[3] there is ample evidence to suggest that either direct surgical intervention with hepatic duct resection and long-term stenting with silastic transhepatic biliary stents, or percutaneous dilatation of strictures, can indeed influence the subsequent course that these patients manifest, if far advanced liver disease is not present. Each series published to date that advocates reconstructive procedures has clearly demonstrated that if cirrhosis is present, nothing less than liver transplantation is likely to help. These patients have end stage disease, and obviously will not benefit from a biliary reconstruction. If one is to influence the subsequent course of sclerosing cholangitis, the intervention obviously has to be performed prior to the development of end stage liver disease. This point is not emphasized adequately in the current manuscript. Another statement the authors make that many might disagree with is that previous surgery on the biliary tract predicts a poor outcome if followed by orthotopic liver transplantation. Even though that appeared to be the case in the current series, in the larger series reported by the Pittsburgh transplant group,[4] patients with sclerosing cholangitis who had been operated upon previously did just as well as those who had not been operated upon. Without question the hepatectomy and transplant are technically more difficult if prior surgery has been undertaken, but ultimate outcome should not be affected. Clearly if a direct biliary tract procedure will prevent the need for liver transplantation, or even if it will delay it for a several year period, it should be performed. That of course is the crux of the argument. Whether or not a procedure performed directly on the biliary tract prior to the development of cirrhosis, will alter the natural history of the disease, and delay or prevent the development or cirrhosis. Data from our unit as well as others suggests that to be the case. The current series, many of whom have end stage disease, cannot answer that question. This series is a large group of ill patients with end stage disease, and the authors are to be complimented on their results with this extremely difficult high risk group.

John L. Cameron
The Johns Hopkins University
School of Medicine
Richard Starr Ross Research Building
720 Rutland Avenue
Room 759
Baltimore
Maryland 21205-2196
United States of America

### REFERENCES

1. Helzberg, J.H., Petersen, J.M. and Boyer, J.L. (1987) Improved survival with primary sclerosing cholangitis. *Gastroenterology*, **92**, 1869–1875
2. Cameron, J.L., Pitt, H.A., Zinner, M.J., Herlong, H.F., Kaufman, S.L., Boitnott, J.K. and Coleman, J. (1988) Resection of hepatic duct bifurcation and transhepatic stenting for sclerosing cholangitis. *Annals of Surgery*, **207**, 614–622
3. Hutson, D., Russell, E., Jeffers, L., *et al.* (1986) Balloon dilation of biliary strictures in primary sclerosing cholangitis through a cholodochojejuno-cutaneous fistula: a new therapeutic approach (abstract). *Gastroenterology*, **90**, 1470
4. Marsh, J.W., Iwatsuki, S., Makowka, L., *et al.* (1988) Orthotopic liver transplantation for primary sclerosing cholangitis. *Annals of Surgery*, **207**, 21–25

### COMMON BILE DUCT STONES : ERCP OR SURGERY?

### ABSTRACT

*Stain, S.C., Cohen, H., Tsuishoysha, M., Donovan, A.J. (1991) Choledocholithiasis . Endoscopic sphincterotomy or common bile duct exploration. Annals of Surgery; 213: 627–634*

**A prospective randomized trial was conducted of preoperative endoscopic sphincterotomy and surgery (ES&S) or surgery alone (SA) in 52 patients with cholecystolithiasis and choledocholithiasis that were candidates for elective surgery. After ES&S**

